# Global Challenges – an innovative journal for tackling humanity's major challenges

**DOI:** 10.1002/gch2.1004

**Published:** 2015-09-21

**Authors:** Fiona G. Hutton, Georg Feulner, Peter D. Lund, Spencer Henson, John‐Arne Røttingen, Steven J. Hoffman, David Butler

**Affiliations:** ^1^ Wiley Open Access, Global Research John Wiley & Sons Ltd Chichester UK; ^2^ Earth System Analysis, Potsdam Institute for Climate Impact Research Potsdam Germany; ^3^ School of Science Aalto University Espoo‐Otaniemi Finland; ^4^ University of Guelph Guelph Ontario Canada; ^5^ Norwegian Institute of Public Health Harvard University and University of Oslo Norway; ^6^ Global Strategy Lab University of Ottawa Ottawa Ontario Canada; ^7^ Centre for Water Systems University of Exeter Exeter UK

## Abstract

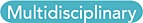

Climate change, energy poverty and security, health security and disease, food and nutrition security, and ensuring safe access to water and sanitation are some of humanity's major challenges. They can lead to poverty and inequality within a world population projected to grow to 9 billion by 2050 and can have a powerful negative impact on economic and social development and on the natural environment. These challenges are also and will increasingly become underlying reasons for injustice, conflicts, and wars. Addressing such challenges in a sustainable manner requires strategic research investments, international collaboration, collective resources, and knowledge exchange between diverse communities. There is increasing awareness that solving such problems requires an interdisciplinary approach: the natural sciences and technology cannot solve these issues alone and must integrate with the social sciences and humanities to identify viable solutions and to ensure that knowledge is informing policy and practice. How communities prioritize research objectives and communicate with one another may determine how effectively humanity can tackle these major global challenges. Our firm belief is that research policy and funding should be heavily influenced towards the global challenges with more interdisciplinary research, multi‐sectorial approaches, cross‐border collaboration, and a clear focus on relevance for national and global policy making leading to the widespread adoption of best practice.

In response, we are excited to announce the launch of *Global Challenges*, Wiley's new premium interdisciplinary open access journal. *Global Challenges* will publish high‐quality research papers, reviews, editorials, and commentaries spanning research and practice related to these global challenges. The aim is to mobilize debate and leadership regarding these challenges and to create a platform for directing and setting the research, policy, and practice agendas. In doing so, we provide a new centre for an emergent community of cross‐disciplinary collaborative stakeholders.

*Global Challenges* will initially focus on five major challenges: Climate Change, Energy, Water, Global Health, and Food, Agriculture & Nutrition, with each subject area overseen by Chief Editors (see accompanying editorials). Each area has an Editorial Board working alongside the respective Chief Editor in order to attract and select the highest quality papers. *Global Challenges* will publish cutting‐edge research, selected through a strict, transparent, and fair reviewing process. We intend to demonstrate to funders that the research they fund has impact, with each research article containing an impact statement and a more detailed impact box. The intention is to put the research into context for a broadly based group of stakeholders. We will also encourage rigorous research synthesis through systematic reviews that can summarize the total body of research on a question with the aim to inform policy and practice in an unbiased manner. In addition, editorials and commentaries will identify priorities both for research and policy and initiate debate on how best to address global challenges.

We are very aware that, to make measurable progress in the mitigation of global challenges, we need to encourage multidisciplinary conversations between scientific fields and between natural scientists, technologists, social scientists, and those in the humanities, not just act as a venue for broadcasting results and findings. We want to advance dialogue and research and enable decision‐makers to base policy and practice on scientific evidence. Our intention is to consult regularly and widely with stakeholders on how best to achieve this aim.

It is important to state that open access plays a central role in this initiative. Funders invest in research to encourage discovery and innovation and to progress economic development. Open access, and therefore open digital knowledge, advances research by maximizing the extensive use and interrogation of data and information, enabling researchers and other stakeholders to deliver an accelerated return on society's investment in research. We should also recognize that open access enables us to reach into communities central to addressing the global challenges, communities not normally exposed to cutting edge research. Practitioners as well as policy makers will be an essential component in driving the debate.

*Global Challenges* will not simply be another journal; it will be a *different* kind of journal, worthy of the best manuscripts. By embracing policy‐relevant work, including applied research and analysis, *Global Challenges* will bridge the typical divide between “science and technology” and “policy and practice” that most current journals impose. By embracing interdisciplinary work, along with the full range of disciplinary traditions, it will be *the* venue for publishing cross‐sectoral research that addresses several challenges simultaneously. It is important to note that the journal is the first building block; our aim is to build on the scope of *Global Challenges* to embrace flexible and innovative communication technologies, enabling us to maximize dissemination reach and influence global events.

We hope you are equally enthused, challenged, and mobilized by this initiative, and we welcome thoughts, input, and suggestions. We also hope you will join us on this mission by bringing your expertise, ideas, opinions, and voice. We want not only to create a journal but also to create a global community with a global mission.

Email us at challenges@wiley.com.

